# Effectiveness of combined chloroquine and primaquine treatment in 14 days versus intermittent single dose regimen, in an open, non-randomized, clinical trial, to eliminate *Plasmodium vivax* in southern Mexico

**DOI:** 10.1186/s12936-015-0938-2

**Published:** 2015-10-30

**Authors:** Lilia Gonzalez-Ceron, Mario H. Rodriguez, Marco A. Sandoval, Frida Santillan, Sonia Galindo-Virgen, Angel F. Betanzos, Angel F. Rosales, Olga L. Palomeque

**Affiliations:** Regional Centre for Public Health Research, National Institute for Public Health, Tapachula, Chiapas Mexico; Centre for Research of Infectious Diseases, National Institute for Public Health, Cuernavaca, Morelos Mexico; Laboratory of Malaria, National Institute for Diagnosis and Epidemiological Reference, Mexico City, Mexico

**Keywords:** *Plasmodium vivax*, Chloroquine, Primaquine, Effectiveness, Single dose, ISD, T14, Microscopy, PCR, Serology, Relapse, Genotype, Southern Mexico

## Abstract

**Background:**

In Mexico, combined chloroquine (CQ) and primaquine (PQ) treatment has been used since the late 1950s to treat *Plasmodium vivax* infections. Although malaria transmission has declined, current treatment strategies must be evaluated to advance towards malaria elimination.

**Methods:**

The clinical and parasitological outcome of treating symptomatic *P. vivax* with the 14-day (T14) treatment or intermittent single dose (ISD) regimen was evaluated in southern Mexico between February 2008 and September 2010. Patients over 12 months old with *P. vivax* mono-infection and asexual parasitaemia ≥500 parasites/µl were treated under supervision. After diagnosis (day 0), treatment began immediately. T14 patients received CQ for 3 days (10, 10 and 5 mg/kg) and PQ daily for 14 days (0.25 mg/kg), while ISD patients received a single dose of CQ (10 mg/kg) and PQ (0.75 mg/kg) on days 0, 30, 60, 180, 210, and 240. Follow-up was done by observing clinical and laboratory (by microscopy, serology and PCR) outcome, considering two endpoints: primary blood infection clearance and clinical response at ~28 days, and the incidence of recurrent blood infection during 12 months. Parasite genotypes of primary/recurrent blood infections were analysed.

**Results:**

During the first 28 days, no differences in parasite clearance or clinical outcome were observed between T14 (86 patients) and ISD (67 patients). On day 3, 95 % of patients in both groups showed no blood parasites, and no recurrences were detected on days 7–28. Contrarily, the therapeutic effectiveness (absence of recurrent parasitaemia) was distinct for T14 versus ISD at 12 months: 83.7 versus 50 %, respectively (p = 0.000). Symptomatic and asymptomatic infections were recorded on days 31–352. Some parasite recurrences were detected by PCR and/or serological testing.

**Conclusions:**

T14 was effective for opportune elimination of the primary blood infection and preventing relapse episodes. The first single dose of CQ-PQ eliminated primary blood infection as efficiently as the initial three-dose scheme of T14, but the ISD regimen should be abandoned. A single combined dose administered to symptomatic patients in remote areas while awaiting parasitological diagnosis may contribute to halting *P. vivax* transmission. Alternatives for meeting the challenge of T14 supervision are discussed.

Trial registration: NIH-USA, ClinicalTrial.gov Identifier: NCT02394197

**Electronic supplementary material:**

The online version of this article (doi:10.1186/s12936-015-0938-2) contains supplementary material, which is available to authorized users.

## Background

According to malaria records, which have existed in Mexico since 1949, the last epidemic occurred in the 1980s. At that time, more than 120,000 cases were reported each year and malaria transmission was present in over 50 % of the country. Due to the intensification of control activities, transmission has now been reduced to a few residual foci. In fact, local transmission of *Plasmodium falciparum* was completely eliminated in 2000, the same year in which 7259 *Plasmodium vivax* cases were reported. The number of cases of the latter *Plasmodium* species was reduced to 2702 in 2009. The country is currently considered to be in the pre-elimination phase [1–4].

In most affected areas around the world, a combination treatment of chloroquine (CQ) and primaquine (PQ) was introduced in the 1950s to treat *P. vivax* blood and liver infections [5, 6]. In regions where parasites are susceptible to these drugs, the recommended doses are the following: CQ at 25 mg/kg bw (body weight) during three consecutive days (10, 10 and 5 mg/kg bw) and PQ at 0.25–0.75 mg/kg bw during 14 days [7, 8]. In fact, this 14-day (T14) treatment effectively cures primary blood infections and is the most effective for preventing relapses [9]. However, in areas where CQ is no longer effective, it is recommended that artemisinin combination therapy (ACT) be used instead, also in combination with PQ for radical cure [8]. No studies exist on CQ resistance in Mexico.

Like most of the Americas, Mexico has been using the combination of CQ and PQ as the standard treatment for *P. vivax* malaria to date. However, PQ treatment is frequently not completed due to its undesirable side effects. Although haemolysis due to glucose 6-phosphate dehydrogenase deficiency (G6PDd) in PQ-treated patients is a concern in malaria-affected regions [8], the Mexican Malaria Control Programme (MCP) has administered 0.75 mg PQ in a single dose for decades without G6PDd testing [10]. In this country, G6PDd occurs in less than 1 % of the general population (the African-related variant is the most common) [11–13].

In locations of difficult access, the intervals between blood sampling, parasitological diagnosis and patient treatment used to be as long as 3–4 weeks. Hence, to reduce the risk of transmission, febrile patients were blood sampled and immediately treated with a single dose of 10 mg of CQ and 0.75 mg of PQ per kg bw [14, 15]. Then, after microscopy confirmation, the full T14 treatment was administered. In 1999, an intermittent single dose regimen (ISD) replaced T14. The ISD regimen, allegedly easy to dispense under field conditions, comprises monthly administration of a single dose for 3 months, followed by 3 months without dosing. This schedule is repeated twice each year to complete 3 years of treatment, with a total of 18 doses [10, 16, 17].

The first single combined CQ-PQ dose is expected to eliminate blood parasites, since PQ may destroy parasites surviving from the effect of CQ [15, 18, 19]. The repeated administration of ISD was intended to suppress cases of recurrent parasitaemia [10, 17]. However, symptomatic recurrent infections in southern Mexico (probably relapses) have been detected within 12 months of the initial treatment [20]. Assessment and monitoring of the effectiveness of T14 and ISD treatment is necessary in order to assess their efficacy and contribution to malaria reduction, with the aim of advancing towards malaria elimination in the region [21]. The aim of the present study was to compare the effectiveness of the standard T14 treatment with that of the ISD regimen in patients living in endemic communities of southern Mexico. Two endpoints were considered: their efficacy in clearing primary clinical and parasitological infections within 28 days post-diagnosis, and in treating recurrent *P. vivax* infections during a 12-month period.

## Methods

The protocol of this study was approved by the Ethics in Research Review Committee of the National Institute of Public Health (Mexico). Informed consent was obtained from all patients or the guardians of minors.

### Study design

An open-labelled, non-randomized, clinical trial was conducted with a group of malaria patients receiving a combined dose of CQ and PQ with either an ISD schedule or the standard 14-day treatment (T14). In both cases, the first dose was given on day 0, the day of diagnosis. The treatment dosing was adjusted according to the age group of a patient, as recommended by the Mexican MCP for ISD and T14 [10]. The study was designed to evaluate two clinical and parasitological endpoints: the primary parasite blood infection clearance and clinical response at ~28 days post-diagnosis, and the incidence of recurrent blood infections during 12 months.

### Study site

The study was carried out in the Tapachula municipality and surrounding communities in southern Chiapas, Mexico, a region on the Guatemala border with an altitude ranging from ~50–1200 meters above sea level. This is a hypo-endemic region showing variable malaria incidence, with an annual parasite incidence (API) of 0.579–1.523 cases/1000 inhabitants when considering only the affected villages for the period 2003–2007 (data from the Local Sanitary Jurisdiction VII). Patient recruitment was carried out in the diagnosis facility at the Regional Centre for Public Health Research-INSP (CRISP) in Tapachula City, where febrile individuals seeking malaria diagnosis and treatment can obtain free service.

### Patients

Patients were recruited from February 2008 to October 2009 and the follow-up ended in September 2010. Inclusion criteria were: (1) patients over 12 months old; (2) *P. vivax* mono-infection, confirmed by microscopy; (3) parasite densities of 500-50,000/μl of blood; and, (4) elevated axillary temperature (≥37.5 °C) or a history of a fever episode within the previous 48 h. Patients were excluded if they: (1) lived in communities that could not be reached by travelling for 1 h by motor vehicle from the CRISP centre; (2) presented signs of severe malnutrition or anaemia; (3) had taken an anti-malaria treatment or had had a malaria infection within the previous 12 months (to reduce the probability of incorporating patients with a relapse) [20]; (4) had another possible cause for their fever or suffered from some chronic disease; (5) were allergic to CQ; or, (6) were pregnant (by patient statement and/or pregnancy test of women over 14) [7]. The pregnancy test consisted of a urine sample evaluated with Gestastrip II (Hycel, Jalisco, Mexico).

For practical purposes, patients were not randomized to form the two treatment groups (ISD and T14). Patients living in Tapachula City and the nearby area were preferred to administer the T14 treatment in order to facilitate daily supervision. On the day of diagnosis (day 0), patients were examined for axillary temperature, body weight, spleen swelling, and clinical symptoms such as fever, headache, myalgia, arthralgia, erythema, and jaundice. Capillary blood, collected by finger pricking, was used to prepare thin and thick smears and to impregnate filter paper (Whatman #2) for parasitological and serological (DNA and antibody) analysis, respectively.

### Patient treatment and follow-up

For the ISD regimen, the standard single dose consisted of 10 mg/kg of CQ and 0.75 mg/kg of PQ, which the patients were scheduled to take on days 0, 30, 60, 180, 210, and 240. For the T14 treatment, the optimal dose was 25 mg/kg of CQ administered over a three-day period (10 mg/kg on days 0 and 1 and 5 mg/kg on day 2) and PQ at 0.25 mg/kg/day given during 14 days [7, 8]. However, both treatment regimens were adjusted to the age group of patients, according to the operational table prescribed by the Mexican official guidelines (Additional file 1) [10]. For small children, tablets were crushed, mixed with water and spoon fed. Treated patients were observed during 30 min after ingesting the drugs. Those who vomited the first dose were re-treated with a similar dose, and those who vomited again were treated, but excluded from the study.

The CQ and PQ tablets were provided by the local malaria control programme (drug batches used during the study are shown in Additional file 2). The first dose was administered immediately after diagnosis at CRISP (day 0), and all subsequent doses were administered under supervision by a member of the study team. If a patient was not available to receive one or two supervised doses, these were left with a family member. If the next day the patient claimed to have taken the dose and was willing to continue with the medication and follow-up, he/she remained in the study within the semi-supervised group.

In case of feeling ill (having fever or presenting one of two other malaria symptoms) at any time during the follow-up period, patients were encouraged to visit the facility at CRISP or to contact the field team by mobile phone (most patients or their parents had a mobile phone). The same recommendation was given in the event that patients had any question about the study. Whether during the treatment or follow-up period, participants unable to reach the facility were visited at their homes for treatment, clinical revision and blood sampling. All treated patients were scheduled for examination on days 2, 3, 7, 14, 21, and 28 [22]. Afterwards, parasitological and clinical examinations were implemented monthly until completing the 12-month protocol. When required, scheduling changes were made to complete the follow-up for each patient, as suggested by Ruebush et al. [23]. If patients, after the first ISD administration, were not at home on day 3, they were visited on day 4. Likewise, visits were made on day 7 ± 1, and on day 14 ± 2. For all patients (under ISD or T14), a visit was carried out on day 21 ± 2, and so on for the rest of the follow-up.

Two thick and thin blood films and a sample of blood impregnated in filter paper (Whatman #2) were prepared at every scheduled or extra visit. Patients with axillary temperatures ≥37.5 °C were symptomatically treated with paracetamol (acetaminophen) (10 mg/kg). A rapid diagnostic test (RDT) OptiMAL© [24] was applied to symptomatic patients when visited at home. Thick smears of symptomatic patients with negative RDT were analysed the same day and if a recurrent *P. vivax* episode was confirmed, they were immediately treated with T14 [8].

### Parasitological diagnosis

Thick and thin blood smears were stained with 10 % Giemsa (pH 7.2) for 5 min. The two thick smears were independently examined under a light microscope by two trained laboratory technicians by using oil immersion 100×. Asexual and sexual parasite densities were determined by counting the number of parasites found per 200 white blood cells (WBC) assuming 7000 WBC/μl of blood [24, 25]. In case of very light infections (less than 10 parasites/200 WBC), 500 WBC were inspected to confirm the counts. At least 500 fields were examined to record a sample as negative. One thick smear was analysed at CRISP and the second one by another microscopist at the Institute of Diagnosis and Epidemiological Reference, the national reference laboratory of quality control for malaria diagnosis in Mexico. Discrepancies on smears results (absence versus presence of parasites or counts differing by 50 %) were resolved by cross validation, examined by a third experienced microscopist. Asexual parasitaemia was quantified using the following formula: parasites/µl of blood = (number of parasites per 200/500 fields × 7000 WBC per 1 µl of blood)/number of WBC per 200/500 fields.

### Molecular diagnosis (PCR)

Molecular diagnosis has proven to be very sensitive in different regions affected by malaria [26, 27]. All blood samples (whether taken on days 2, 3, 7, 14, ~21, or ~28, or thereafter during the monthly examinations) with an increase in the anti-*P. vivax* IgG antibody titre (ELISA OD value), were subjected to molecular diagnosis due to suspicion of recent malaria infection. Six 5-mm punches of filter paper impregnated with dried blood were used to extract DNA, using the QIAamp^®^ DNA Blood Mini Kit (QIAGEN, Hilden, Germany) and following the manufacturer’s instructions. The DNA obtained was suspended in 50 µl of water and stored at −20 °C until used. The presence of the *P. vivax* 18S small sub-unit ribosomal RNA gene was assessed as previously reported [28]. Infected (with *P. vivax*: 500, 2000 and 10,000 p/µl) and uninfected blood samples were included as controls.

### Detection of IgG antibodies against the *Plasmodium vivax* blood stage (by ELISA)

Antibodies against the *P. vivax* blood stage can last for months or years in exposed patients. However, antibody titres fade out more rapidly in treated patients [29–31]. To reveal *P. vivax* re-exposure, with and without clinical symptoms and/or low parasitaemia in recurrent blood infections, retrospective research was conducted at the end of the 12-month follow-up period to look for native *P. vivax* blood-stage proteins in preserved blood samples by using ELISA, as previously reported [32]. Briefly, the blood samples that had been taken from each patient and smeared on filter paper were eluted in PBS and tested at 1:500 dilutions in an indirect ELISA. The reaction was revealed using goat anti-human IgG (H + L)-HRP (Pierce, Rockford, IL, USA) diluted 1:5000 and ABTS (2,2′-Azinobis [3-ethylbenzothiazoline-6-sulfonic acid]-diammonium salt) as substrate for 60 min, and then OD values were recorded in a spectrophotometer (Biotek^®^ model EL312) at 405 nm. Cut-off values were previously determined using unexposed individuals as the mean and 2SD as 0.25 OD values (95 % confidence). The ELISA-OD values were plotted according to time point per patient.

### Primary and recurrent *Plasmodium vivax* genotype determination

To determine the frequency of parasite genotypes, all *P. vivax* infected blood was analysed for *cspr* and *msp3α* by polymerase chain reaction and restriction fragment length polymorphism (PCR–RFLP) [20]. The homogeneity of *P. vivax* genotypes among groups was examined by Fisher’s exact test (α = 0.05). In addition to *cspr* and *msp3α*, parasite genotypes were analysed using *msp3β* after *Alu I* digestion to compare parasites producing primary and secondary episodes in each patient [33].

### Data analysis

For each patient that completed the supervised treatments and the follow-up (for 28 days and 12 months post-diagnosis), parasitological and clinical data were analysed using STATA v12. To discard the possibility that a sub-dose caused delay in parasite clearance, the body weight recorded on day 0 was used to determine the accuracy of doses of CQ and PQ given to patients using age group dosing.

The first endpoint, parasitological and clinical cure of the primary blood infection, was evaluated by determining the early clinical and parasitological outcome at 2–3 days post-diagnosis, and the possible clinical and/or parasitological asexual parasite persistence or reappearance within seven to 28 days post-diagnosis. Therapeutic failure (TF) was defined as the presence of parasitaemia, while an adequate clinical and parasitological response (ACPR) was considered with the absence of parasites on days 7–28: for these analyses, both microscopic and PCR results were included.

Fisher’s exact test was used to compare two or more independent proportions between treatment groups, considering gender, PCR positivity, parasite persistence, the presence of symptoms, and the distribution of the *P. vivax* genotype on day 2 or 3. The Wilcoxon rank sum test was used for non-parametric comparisons: age, asexual and sexual parasitaemia, drug doses, number of days of symptoms, and axial temperature. All tests of significance were two-tailed and *P* values < 0.05 were considered statistically significant.

The second endpoint evaluated was by determining whether or not recurrent blood infections existed during the follow-up ending 12 months after day 0. The effectiveness was calculated as the per cent of the accumulated number of patients without *P. vivax* blood infection (first recurrent case) detected at each month of sampling, divided by the total number of patients followed up. The Z-test was used to determine differences in the proportion of patients or samples with a recurrent infection. To plot the cumulative incidence of patients with the first recurrence, the Kaplan–Meier failure estimate was used.

## Results

### Demographics, clinical and parasitological characteristics of patients

**Fig. 1:**
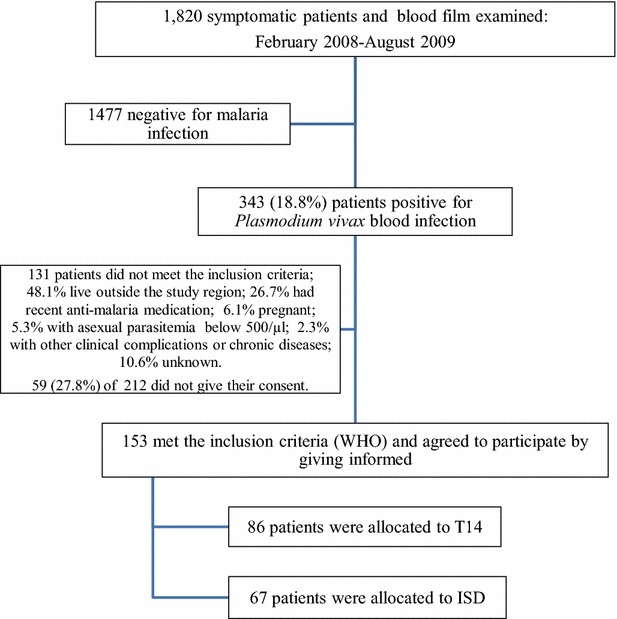
Flow chart of *Plasmodium vivax* patient detection and recruitment for T14 and ISD treatment regimens, in southern Mexico. *D* day

During the recruitment period, the diagnostic facility at CRISP received 1,820 symptomatic patients, of which 343 were diagnosed with *P. vivax* by microscopy. Of the 153 self-reporting malaria-symptomatic patients that satisfied the inclusion criteria, 86 received the T14 treatment and 67 the ISD regimen (Fig. 1). More patients were assigned to T14, expecting that several patients would not complete all supervised T14 doses. The proportion of gender and median age of participants, median and interquartile range (IQR) of the duration of symptoms (in days), and parasite densities were similar in both groups (Table 1). There was no difference in the proportion of patients indicating certain malaria symptoms, such as fever, headache, myalgia, arthralgia, chills, jaundice, blisters, and erythema (Table 1).

**Table 1:** Characteristics of patients participating in the study: CQ–PQ combined treatment in 14 days (T14) and intermittent single doses (ISD)

Parameters	By regimen of anti-malarial treatment				*P* value
	Single doses (ISD)		14-day (T14)		
Total patients	67		86		
By Gender (n)					
	Female	Male	Female	Male	*0.274* ^a^
Age range in years					
1–5	2	3	2	3	Age by gender (male/age, *0.58*; female/age, *0.413*)
6–15	13	6	6	13	
16–25	6	6	9	9	
26–40	4	6	9	12	
>40	9	12	10	13	
Total	34	33	36	50	
Body weight: median (IQR), range	50 (25), 9.6–108		57 (20), 14–104		*0.219* ^b^
Days of symptoms: median (IQR), range	5 (4), 1–20		4 (4), 1–30		*0.265* ^b^
Axial temperature (°C): median (IQR), range	37.45 (1.1), 36–40		37.5 (1), 36.5–40.1		*0.183* ^b^
Asexual parasitaemia/µl: median (IQR), range	4888 (5379), 512–28,940		3830 (5443), 490–28,424		*0.20* ^b^
Samples with gametocytes	100 %		100 %		*0.073* ^b^
Gametocytaemia/µl: median (IQR), range	697 (959), 45–5684		999 (1305), 35-12,102		
Clinical symptoms indicated by patients as having occurred within 2 days of arrival to our diagnosis facility (% of patients indicating each condition)					*0.265* ^c^
Fever	98.1		100		
Headache	98.1		96.5		
Mialgias	89.5		89.5		
Arthralgias	80.6		83.7		
Paroxysm	77.6		68.6		
Jaundice	11.9		12.8		
Blisters	2.9		3.4		
Pruritus	0		0		

*F* female, *M* male, *n* number of patients, *IQR* interquartile range

^a^Chi square; α = 0.05

^b^Two-sample Wilcoxon rank-sum (Mann–Whitney) test

^c^Fisher exact test; α = 0.05

Most patients recruited for the study were living in an area of 18 × 10 sq km and they were comprised of mostly indigenous and Mexican mestizo populations. Although a higher proportion of T14 patients lived in the Tapachula City and its surrounding areas (zone 1), and a higher proportion of ISD patients lived in the foothills (zone 2) (Additional file 3), ISD and T14 patient groups had similar proportions of *P. vivaxcspr*-*msp3α* genotypes (*p* = *0.248*; Chi square test; Additional file 4). There was a significantly lower CQ dose in male patients when comparing the ISD group (median dose 0.88/IQR 0.16) to the T14 group (median dose 1.0/IQR 0.29, *p* = *0.01*).

### Clinical and parasitological clearance of the primary blood infection during 28 days

Out of 67 participants that received the initial dose of ISD, 18 (26.8 %) and 23 (34.3 %) completed the programmed follow-up visits and the flexible-schedule visits, respectively. Patients missing three continuous scheduled visits were excluded from the follow-up. Thirteen patients were not accessible for one visit during the first 14 days, and another four missed two follow-up visits. There were nine patients who withdrew, including four on day 2, two on day 3, one on day 7, and two on day 14. Six patients were not willing to be subjected to finger pricking, two moved to inaccessible communities, and another took an extra medication of CQ-PQ on day 3.

Out of 86 patients with the T14 treatment, 49 (56.9 %) completed the 14 supervised doses. Of these, 44 completed all follow-up visits (24 as programmed and 20 with the flexible schedule option, needed only for days 21 and 28). Three patients missed one visit, on day 21 or 28. There were two withdrawals of patients afraid of finger pricking on day 21.

### Comparison of parasite detection by microscopy and PCR

A total of 778 samples obtained from days 2 to 28, examined by both microscopy and PCR (including three samples with only gametocytes), were used to compare the performance of the diagnostic methods. Of these, 90.3 % had concordant results (35 were positive and 703 negative for *P. vivax* according to both methods). There were 32 samples (4.1 %) only PCR-positive, indicating that parasite density was at the sub-microscopic level. Eight samples (1.22 %) were microscopy-positive but PCR negative. These discordant samples had parasite densities below 100/μl (16, 28, 28, 49, 70, 82, 88, and 197 p/μl). Three samples (with parasite densities below 100/μl) were positive in only one of the two thick smears.

### Early clinical and parasitological responses (on days 2 and 3)

Of the T14 group, 81 and 75 were sampled during supervised treatment on day 2 and 3, respectively. For the ISD group, 57 patients were sampled on these 2 days. There were no differences in the clinical outcome between the two treatment groups (Table 2). In seven of these patients (three in the T14 group and four in the ISD group), small blisters on the lips and/or outer edges of the mouth were detected. In most of these patients, temperatures above 38 °C had been registered at recruitment/diagnosis.

**Table 2:** Clinical and parasitological outcome of *Plasmodium vivax* affected patients treated with combined doses of CQ–PQ for 14 days (T14) or with an intermittent single dose (ISD)

N Days (mean/SD)	57^e^ (2/0)	59^e^ (3/0.15)	54 (7.2/0.74)	54 (14.6/1.1)	56 (21.4/1.2)	60 (29.8/2.9)
Single dose						
Clinical outcome (% with symptoms)						
Fever	10.17^†^	3.70^†^	1.8^†^	0	0	0
Headache	30.51^†^	16.67^†^	3.7^†^	5.3^†^	3.3^†^	0
Mialgias	1.69^†^	3.39^†^	0	0	0	0
Arthralgias	0	0	0	0	0	0
Blisters	5.08	6.78	7.41	3.7	1.8	0
Erythema/pruritus	10.17	8.47	1.85	0	0	0
Light jaundice	11.86	13.56*	11.11*	9.2*	3.6	0
Asexual parasitaemia by microscopy						
Positivity: n, %^a^	14, 23.7	3, 5.0	0	0	0	2, 3.4
p/µl^b^: median (IQR)^c^	62 (68)	157.7 (59.1)	–	–	–	223 (163)
% PCR positive^d^	41.3	12.7	0	0	0	3.4

N Days (mean/SD)	81 (2)	75 (3)	63 (7)	49 (14)	44 (21.5/1.1)	44 (29/2.5)
14-day (T14)						
Clinical outcome (% with symptoms)						
Fever	2.67^†^	3.17^†^	0	0	0	0
Headache	25.33^†^	7.94^†^	0	2.2^†^	0	0
Mialgias	2.47^†^	4.0^†^	0	0	0	0
Arthralgias	1.23	1.33	0	0	0	0
Blisters	3.70	4.0	3.17	0	0	0
Erythema/pruritus	7.41	9.33	6.35	4.1	0	0
Light jaundice	23.46	32.0*	36.5*	40.8*	13.6	2.2
Asexual parasitaemia by microscopy						
Positivity: n, %^a^	13, 16	4, 5.1	0	0	0	0
p/µl^b^: median (IQR)^c^	118 (180)	42.4 (43.0)	–	–	–	–
% PCR positive^d^	34.1	8.75	0	0	0	0

*N* number of patients

* Statistical differences were detected on days 3 (*p* = *0.004*), 7 (*p* = *0.007*) and 14 (*p* = *0.002*) in both treatment groups (Fisher exact test, α = 0.05)

^†^ As indicated by the patients, this symptom was present between visits

^a^n and %, number and percent of samples with asexual parasites

^b^Number of asexual parasites per microlitre of blood

^c^Only positive samples were included

^d^Percent of samples positive by molecular analysis

^e^Patients sampled on day 2 were not sampled on day 3 and vice versa

No difference in parasitaemia clearance was observed between treatments. Few patients had asexual blood parasites detected by microscopy on days 2 or 3 (Table 2). The reduction in the number of patients with asexual parasitaemia on day 3 with respect to day 2 was significant and similar for both treatments (T14 *p* = *0.000*; SD *p* = *0.016*). The number of PCR-positive samples on days 2 and 3 was higher than that determined by microscopy. On day 2, 41.3 % (1.87-fold higher than microscopy) and 34.1 % (2.3-fold higher than microscopy) of samples were PCR-positive for the ISD and T14 treatments, respectively. Persistent parasitaemia, detected by microscopy and/or PCR on days 2 and 3, was inversely correlated to the parasite count on day 0. That is, a higher parasite density (median 5068/IQR 2,067) was found on day 0 in patients that earlier cleared blood parasites, compared to the density observed (median 2891/IQR 1,357, also on day 0) in patients that on days 2 and 3 still harboured blood parasites (*p* = *0.0375*). On the other hand, parasite persistence was not associated with parasite genotype, lower CQ doses, or patient gender or age.

### Treatment failure (days 7–28)

All samples from days 7 to 21 were negative by microscopy and PCR, regardless of the treatment (Table 2). A light yellowish jaundice was observed in some ISD and T14 patients. In some ISD patients, this symptom persisted through day 14, while among T14 patients, the proportion with light jaundice increased with PQ administration (representing 40 % on day 14). No patient reported dark urine or any other clinical symptoms of haemolysis. In all patients, the jaundice disappeared by day 28, and no additional treatment was necessary. Erythema, present in some patients of both treatment schedules, disappeared after completion of the treatment. Light jaundice, erythema and/or blisters occurred concomitantly in only three patients.

Light jaundice in T14-treated patients was associated with the number of days with malaria symptoms before recruitment. The group with light jaundice had a higher proportion of patients with more symptomatic days previous to recruitment (median of days with symptoms, 16.5/IQR 2), compared to patients with no jaundice (median of days with symptoms 14/IQR 4; *p = 0.0067*). The presence of these symptoms was not associated with parasitaemia levels on day 0, parasite persistence (observed on days 2 and 3), gender, or PQ dosage.

### T14 and ISD: prevention/elimination of recurrent blood infections (*Plasmodium vivax*) during 12 months

To compare the frequencies of recurrent infections for the T14 and ISD groups, only patients that completed at least 3 months of follow-up were included. Thirty-seven and 49 patients under supervised T14 and ISD treatment, respectively, met this criterion. The number of recurrent infections that occurred in patients recruited to T14 and ISD groups was truthful; the recurrences  were not associated with a particular geographic location (Additional file 5). Moreover, no differences were observed between the 18 patients that completed the semi-supervised T14 treatment and those whose doses were all supervised. All samples from these 18 patients (101 blood samples obtained from day 3 to 28) were negative by microscopy and PCR, similar to the supervised group.

**Fig. 2:**
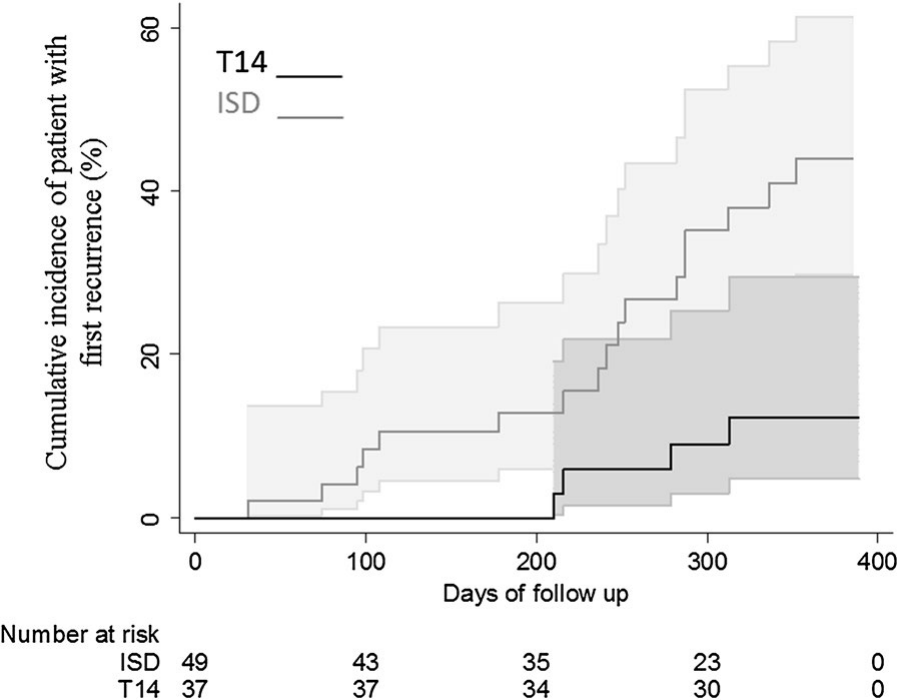
Cumulative incidence of *Plasmodium vivax* recurrences during the 12-month follow-up in groups of patients treated under supervision with combined CQ–PQ for 14 days (T14) and intermittent single doses (ISD). The CI at 95 % is shown

The comparison between the number and timing of recurrent blood infections in the T14 *versus* ISD treatment group is shown in Table 3 and Fig. 2. Three patients (P3, P47, P57) under supervised T14 had a symptomatic recurrent blood infection, after asymptomatic periods of about 7–8 months (late recurrences). All these patients were affected by different *P. vivax* genotypes, but the genotypes of parasites during relapses were similar to those of their primary infections (homologous recurrent infections) (Table 4). Similarly, another three patients under semi-supervised T14 (P70, P113, P149) had late symptomatic recurrent blood infections (detected by microscopy) that were genetically homologous to their primary infection. No difference in the proportion and timing of recurrent episodes between supervised versus semi-supervised T14 patients was identified (*p* = *0.54*; Additional file 6). A significantly higher number of recurrent blood infections was detected in patients under the ISD regimen (Z value 3.85; *p* = *0.000*) (Table 3). Eighteen of the 49 patients in this group had at least one recurrent blood infection, detected by microscopy and/or PCR. The majority of the recurrent episodes were symptomatic (17 of 23), whether confirmed by microscopy and/or PCR (Table 5; Additional file 7). Upon confirmation of the first symptomatic recurrent infection, patients were immediately treated with T14. Because blood samples in asymptomatic patients were not processed immediately after sampling, some cases of recurrent parasitaemia were not opportunely detected. The recurrent cases in this group were distributed at similar frequencies of 23.3, 20, 30, and 27.7 % within the first, second, third, and fourth quarters of the year. Only 33 % (13 of 29) of recurrent blood infections coincided with the administration of the scheduled ISD dose.

**Table 3:** Monthly effectiveness of T14 and ISD treatments to eliminate *Plasmodium vivax* recurrent infections in patients of southern Mexico

A. T14; N = 37												
Day of sampling (mean/SD)	29/3	59/8	92/5	124/4	152/6	185/5	214/5	247/4	277/6	305/7	336/6	370/11
n, samples	35	33	34	30	28	28	28	25	23	25	23	27
n 1rst *P. vivax* recurrence	0	0	0	0	0	0	2	1	0	1	0	0
Cumulative lost	0	0	0	0	0	1	3	4	4	4	5	6
% Effectiveness	100	100	100	100	100	100	94.1	90.9	90.9	87.8	87.5	87.1
B. ISD; N = 49												
Day of sampling (mean/SD)	29/3^a^	62/4^a^	93/6	121/8	154/6	187/7^a^	218/7^a^	246/7^a^	278/11	306/12	336/10	367/22
n, samples	49	48	46	42	40	39	34	28	24	24	22	20
n, 1rst *P. vivax* recurrence	2	1	2	1	0	1	1	3	3	2	1	1
Cumulative lost	0	0	0	2	2	6	9	12	12	12	12	13
% Effectiveness	95.9	93.8	89.7	87.2	87.2	83.7	80	70.2	62.1	56.7	54	50
*P* value^b^	*0.214*	*0.126*	*0.045*	*0.023*	*0.023*	*0.011*	*0.076*	*0.031*	*0.005*	*0.003*	*0.002*	*0.001*

Only patients that completed at least 3 months of follow up were included in this table; four patients under T14 and 18 under ISD had at least one recurrent blood infection; a recurrent blood infection was diagnosed by RDT and/or microscopy or PCR (only two patients; P45 and P63)

Percentage effectiveness was calculated as the number of patients with no malaria infection divided by the total of patients under follow-up

*N* number of patients, *SD* standard deviation

^a^ISD was provided

^b^Z-test, comparing T14 and ISD, showing the proportions of patients with recurrent infections, at 95 % confidence

**Table 4:** Parameters of primary and recurrent *Plasmodium vivax* blood infections from T14 patients

Patient	Age/sex	Primary blood infection		Recurrent infection by microcopy					Serology (ELISA OD value/405NM)	mg/kg bw administered	
		Asexual parasitaemia (p/µl)	RFLP: *cspr*-*msp3α/β*	Day^a^	Presence of clinical symptoms	Asexual parasitaemia: (p/µl)		Genotype *cspr*-*msp3α/β*		CQ	PQ
P3	24/M	3177	vk210-B/III	D210	Yes	9327^b^	vk210-B/III		0.12_D183_ → 0.62 _D210_	25.86	3.62
P47	14/M	2119	vk210-A/I	D249	No	Mc-PCR-	–		0.08_D187_ → 1.12 _D249_	30.0	4.2
				D279	Yes	3845^b^	vk210-A/I		→0.75_D279_		
P57	50/M	2784	Vk210-K/IV	D216	Yes	2706^b^	vk210-K/IV		0.324_D90_ → 1.405_D216_	21.42	3.0
P61	21/F	836	Vk210-A	D95	No	Mc-/PCR−	–		0.49_D65_ → 1.67_D95_	28.84	4.03
				D313	No	Mc-/PCR+	–		→1.27_D313_		

*M* male, *F* female, *RFLP* restriction fragment length polymorphism, *cspr* circumsporozoite repeat type, *msp3α* merozoite surface protein 3α/β

^a^Day of visit/sample and diagnosis

^b^RDT positive and T14 given; *Mc* microscopy, *PCR* molecular diagnosis; recommended treatment: a total dose of 25 mg CQ and 3.5 mg PQ/kg bw; *bw* body weight

**Table 5:** Parameters of primary and recurrent *Plasmodium vivax* blood infections from ISD patients

Patient	Age/sex	Primary blood infection		Recurrent infection by microscopy				Serology (ELISA OD value/405NM; cut off value 0.25)
		(p/µl) asexual parasitaemia	RFLP: *cspr*-*msp3α/β*	Day^a^	Clinical symptoms	(p/µl) asexual parasitaemia	Genotype *cspr*-*msp3α/β*	
P6	3/F	2364	vk247-A	D241^b^	Yes	875	vk247-A	0.16_D211_ → 2.57 _D241_
P7	17/F	10,766	vk210-B/III	D236^b^	Yes	14,218	vk210-B/III	0.04_D218_ → 1.49 _D236_
P15	62/F	675	vk210-B/IV	D312^b^	Yes	246	vk210-B/IV	0.29_D301_ → > 3.0_D312_
P17	36/F	480	vk210/247-B/III	D108^b^	Yes	8485	vk247-A/I	0.18_D89_ → 2.1_D209_
P35	20/F	2241	vK210-C/V	D336^b^	Yes	846	vK210-C/V	0.18_D308_ → 1.88_D336_
P37	9/M	17,598	vK210-C/V	D287^b^	Yes	866	vK210-C/V	0.16_D287_ → 1.67_D308_
P44	60/M	2951	vk210-C/VI	D282^b^	Yes	844	vk210-C/VI	0.18_D244_ → 0.53_D282_
P56	42/F	7960	vk210-C/V	D178^b^	Yes	1570	vk210/247-A/I	0.41_D147_ → 1.12_D178_
P62	26/M	4319	vk210/247-C/II	D216^b^	Yes	2594	vk210-C/II	0.15_D183_ → 2.48 _D216_
P72	12/M	8059	vk210-B/III	D98^c^	Yes	3505	vk210-B/III	0.963_D92_ → 2.03_D98_

Truncated (complete information is given in Additional file 6)

*M* male, *F* female, *RFLP* restriction fragment length polymorphism, *cspr* circumsporozoite gene central repeat type, *msp3α* merozoite surface protein 3α/β genes

^a^Day of sample collection

^b^After diagnosed with *P. vivax*, T14 was administered

^c^Continued the ISD treatment; P44, was the only patient that had a second recurrent episode after T14 was administered; D348 was positive by microscopy

For 16 of the 20 samples from ISD patients in which the *P. vivax* genotype could be obtained, the primary blood infections matched their respective recurrent parasites (Table 5; Additional file 7). No association existed between the genotype and the time interval between primary and recurrent infections. That is, five patients having a primary infection with genotype *cspvk210*-*msp3α C/msp3β V* relapsed after quite different time intervals (days 32, 66, 95, 178, 287, and 352), and three patients with genotype *cspvk210*-*msp3α B/msp3 β III* also relapsed (with homologous parasites) after distinct intervals (days 74/183, 98 and 236). With all recurrent infections treated with supervised T14, the patients had no further detectable recurrent infections, with one exception (patient P44, 65 days after this treatment).

### Serology

**Fig. 3:**
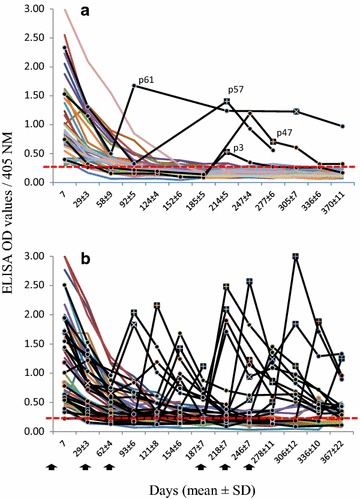
IgG antibodies against the *Plasmodium vivax* blood stage in blood samples. **a** 37 patients treated with T14 and **b** 49 patients under ISD, during the 3–12 month follow-up. Most patients had positive ELISA OD values on day 7 after the initial treatment. Following treatment, OD values decreased or were negative, except in patients with recurrent blood infections. *Squares* with (+) indicate symptomatic *P. vivax* blood infections. *Square* with (×) indicate asymptomatic *P. vivax* that were microscopy and/or PCR-positive. The ELISA OD cut-off value is indicated with a *hidden red line*

 IgG antibodies against the *P. vivax* blood stage were detected by ELISA in 151 of 153 samples, with OD values from 0.3 to >3.0 (mean 1.14 ± 0.73) on day 7 after the initial treatment. Antibody OD values decreased post-treatment, causing nearly 50 % of the patients to be ELISA-negative within 3 months. The antibody response in two patients of the ISD group remained positive, with low OD values, until the end of the study. However, there were no clinical symptoms of a recurrent blood infection or parasitaemia (Fig. 3).

In the T14 group, recurrent *P. vivax* blood infections were accompanied by a drastic increase in the antibody OD value, which decreased after the infection was treated with T14 (Fig. 3a). Antibody titres increased prior to the onset of clinical symptoms and/or parasitaemia. In addition, one patient (P61) likely presented asymptomatic recurrent infections, since an increased and persistently high level of antibody titres was detected on day 95–313. The sample on day 313 was also PCR-positive (Fig. 3a; Table 4). Patient P149 (semi-supervised group) had high antibody titres on days 307 and 322 (on the latter day parasite infection was detected by PCR), suggesting a long period of asymptomatic blood infection. In the ISD group, antibody responses against symptomatic and asymptomatic recurrent infections were observed (Fig. 3b; Table 5; Additional file 7), but in three patients with increased antibody OD values (P22, 0.186_D315_ **→** 0.57_D341_; P87, 0.55_D121_ **→** 1.73_D149_; P115, 0.12_D115_ **→** 0.90_D158_), it was not possible to demonstrate malaria blood infection by microscopy or PCR. No correlation was documented between the low PQ dosage in T14 or ISD and the presence of recurrent blood infections (Table 6). Interestingly, subjects affected with genotype *cspvk247*-*msp3α/A* had a serological response before their recurrent parasitaemia was detected (T14 patients P47 and P61; ISD patients P45 and P80).

**Table 6:** Comparison of patients with or without *Plasmodium vivax* recurrent blood infection and the doses of CQ and PQ managed

Treatment regimen	Patients with recurrent *P. vivax* infection:	n	Total CQ dose/kg Median(IQR)	Total PQ dose/kg Median(IQR)
ISD	No	28^a^	9 (8.18–10)	0.625(0.56–0.72)
	Yes	18	9.91 (9–15)	0.69 (0.6–1.0)
	*P* value^b^	*0.069*		*0.107*
T14	No	33	26.4(24–29.4)	3.75 (3.33–4.2)
	Yes	4	27.3 (23.6–29.4)	3.82 (3.31–4.11)
	*P* value^b^	*0.941*		*0.941*

*n* number of patients, *CQ* chloroquine, *PQ* primaquine

^a^Three patients were not included, since after treatment they had an increase in antibody titres but there was no parasite detected by microscopy or PCR; recommended total doses: for ISD, CQ 10 mg/kg and PQ 0.75 mg/kg bw; for T14, CQ 25 mg/kg and PQ 3.5 mg/kg bw

^b^Wilcoxon rank-sum (Mann–Whitney) test at 95 % CI

## Discussion

The current findings demonstrate a lower performance in preventing relapses exerted by the ISD regimen compared to the T14 standard treatment. Nonetheless, the first ISD single dose (CQ 10 mg and PQ 0.75 mg/kg bw) effectively cleared the primary *P. vivax* parasitaemia, in a similar way as the first three doses of T14. The effectiveness of a single dose in clearing primary blood infections, reported earlier in El Salvador [34], Nicaragua and Costa Rica [14], could support the strategy (followed by the Mexican MCP) of giving an initial single CQ-PQ dose to patients suspected of having malaria until the parasitological diagnosis is completed. A rapid parasite clearance after the initial dose might contribute to reducing transmission, and therefore lead to a continuous reduction of malaria cases in Mexico.

The efficacy of a standard 25 mg/kg bw CQ dose to cure *P. vivax* infection has been proven in many different geographic sites, including Korea [35], Colombia [36], Peru [37], India [38], Pakistan [39], China [40], Thailand [41], Afghanistan [42], Vanuatu [43], Iran [44], Ethiopia [45], and Mauritania [46]. A similar clinical and parasitological outcome was found in Brazil when CQ only was administered at 10 or 25 mg/kg bw [47]. No data on the in vivo effectiveness of CQ are available in Mexico or any other country in the Americas apart from Brazil, and no *P. vivax* resistance to CQ has been evidenced. In this region, a combined regimen of CQ and PQ to treat malaria was introduced in the 1950s and is still effective today, probably because PQ can effectively eliminate parasites with low sensitivity to CQ [8].

Primaquine is the only licensed 8-aminoquinolone available to malaria control programmes for the elimination of liver-dwelling hypnozoites. If left untreated, hypnozoites can produce relapses upon reactivation at variable periods of time [8, 48, 49]. However, PQ-inducing acute haemolytic anaemia (AHA) in G6PD deficient individuals remains an undeniable thread [50, 51]. Thus, PQ administration is in general not recommended before G6PD testing. Nevertheless, PQ-induced AHA depends on the severity of G6PDd, which differs among the more than 180 G6PD variants and in accordance with the PQ dose administered [52]. Low PQ doses can be safely administered, and under field conditions where no G6PD tests are available, the World Health Organization has recommended a single low PQ dose (0.25 mg/kg) to eliminate *P. falciparum* gametocytes [8]. In Mexico, less than 1 % of the general population is G6PD deficient [11–13], and the MCP has been administering 0.75 mg/kg bw PQ in a single dose in the absence of G6PD testing, without any serious AHA symptoms having been identified.

In this study, it is likely that most cases of light jaundice, detected in about 12 % of the patients recruited, were due to infected erythrocyte lysis, and others resulted from PQ administration. The condition of light jaundice occurred mostly in patients under T14 treatment. Indeed, as this treatment progressed there was a greater frequency of jaundice. Although no severe AHA was observed and light jaundice did not worsen in the participants, the current findings warrant further studies to determine G6PDd in the Mexican population affected by malaria infection, as well as its implications for treatment of the same. G6PDd screening would be facilitated by the development of diagnostic tests applicable in field conditions [53].

Mild pruritus was observed in 10 % of the patients in both treatment groups after the administration of CQ–PQ, a condition that disappeared within 1 week. CQ-related pruritus has been reported in a high proportion of Africans, in 1.9 % of Asians [54], and in Brazil [55]. Higher doses of CQ can produce paresthesia, diarrhoea, nausea, and vomiting [56], conditions not seen in patients of the present study.

Although many recurrent episodes were detected by microscopy, the serological analysis of the IgG antibody response to the *P. vivax* blood stage as well as the PCR diagnosis were powerful for discovering recurrent parasite exposure and/or infections, albeit retrospectively. The serological test was very sensitive to antibody titres, which increased after re-exposure to parasites (due to recurrent parasitaemia), as confirmed by PCR. Accordingly, the serological follow up confirmed parasite clearance and the absence of new blood infection. It is possible that there were some recurrent infections cleared without treatment, as evidenced by the antibody response. This situation was reported in Peru [57].

Relapse episodes represent the most important difficulty that impedes the elimination of *P. vivax* transmission [58]. Early relapses can appear in tropical regions as soon as 2 weeks post-primary blood infection [49, 59–61]. Although the blood level of CQ was not measured in this study, other reports have estimated that CQ therapeutic concentrations may remain for 20–35 days after treatment, thus suppressing early relapse episodes [58, 62–64]. In ISD patients, the first recurrent blood infections were detected on days 31 and 32, when CQ might have been at sub-therapeutic levels. The consideration of these episodes as relapses is supported by the fact that these patients had low recurrent parasitaemia and no parasites were detected, either by microscopy or PCR, seven to 21 days post-diagnosis. In T14 patients, the first recurrent blood infections were found on or after day 28 post-diagnosis. Other studies, such as those conducted in Pakistan [39] and Afghanistan [42], are also in accordance with the idea that most recurrent blood infections with *P. vivax* occur within 1 year of the primary infection. The low transmission rate in southern Mexico [65] makes re-infection by an infective mosquito bite unlikely. If in fact most recurrent cases found herein can be attributed to a relapse from hypnozoites in the liver, they should be caused by genetically homologous parasites [20]. Accordingly, in the present study most of the recurrent infections had the same genotype as that of the primary infection. On the other hand, there was no correlation between genotype and relapse latency.

The lack of effectiveness of the ISD regimen was evidenced by the fact that relatively few of the recurrent blood infections (33 %) were suppressed by the programmed single doses. Moreover, as these patients developed parasitaemia they might have been involved as sources of infection before the ISD was administered. The time interval from the primary to the first short- and long-term recurrent infections, as well as the overall pattern of re-infection, partially resembles the outcome reported in Central America, where *P. vivax* strains produced higher numbers of long-term relapse episodes. Worldwide, the T14 treatment has proven to be the most effective treatment for eliminating *P. vivax* relapses [9]. Since the PQ treatment was introduced in Mexico in 1959, the difficulty of completing a 14-day treatment has represented the main hurdle for malaria control. The attempts to reduce this scheme to a 5-day treatment resulted in a higher incidence of relapse [66]. Similar to India [48] and Colombia [67], in Mexico relapse episodes have occurred after short- and long-term periods of latency [20]. In the current contribution, T14 could eliminate almost 100 % of relapses occurring within 6 months and 87 % of those appearing in 12 months. T14 was less effective in Colombia, eliminating 87.4 % of relapses occurring within 4 months [68] and 85 % within 6 months [69] after treatment. In the present study, each patient with a long-term recurrent episode received the appropriate dose of PQ for his or her body weight. Therefore, relapses in these patients appear to be due to problems in drug absorption or low PQ metabolism, as observed in other regions [70, 71].

Although the efficacy of ISD for eliminating primary infections is herein documented, relapses occurred unpredictably at any time within 12 months after the first dose. This treatment scheme has been abandoned by the Mexican MCP. The present pre-elimination status of Mexico requires new strategies for the most thorough detection and treatment of cases [72]. Although the usefulness of the more efficacious T14 might be constrained by its low patient compliance, 59 % of the recruited patients complied with the supervised T14 treatment. Patients with semi-supervised regimen (with family supervision) showed similar outcomes. Hence, this alternative would increase the number of patients receiving effective treatment, and to achieve elimination of malaria in Mexico and other sites with similar epidemiological characteristics. In addition to flexibility in dose scheduling, another factor that could improve malaria control programmes is the determination of the appropriate adjustments to PQ dosing [72], which should be the subject of future studies.

## Conclusions

The first dose of CQ-PQ in the ISD regimen effectively cleared primary clinical and parasitological *P. vivax* blood infections, in a similar way as the initial three doses of the T14 scheme. A single combined dose administered to symptomatic patients in remote areas while awaiting parasitological diagnosis may contribute to halting *P. vivax* transmission. The supervised T14 treatment was much more effective for preventing recurrent infections. Moreover, the semi-supervised T14 (with family supervision) was also effective for patients that did not strictly comply with the supervised scheme. On the other hand, it is clear that at the individual level, ISD scored very low compared to T14 in preventing relapses and should therefore be abandoned. Further research should be conducted to determine the ideal PQ dosing.

## Additional files

10.1186/s12936-015-0938-2 *Plasmodium vivax* treatment regimens based on chloroquine and primaquine, by age groups as indicated in the Mexican guidelines for malaria treatment.
10.1186/s12936-015-0938-2 Chloroquine and primaquine drug batches used during the study.
10.1186/s12936-015-0938-2 Study site and geographic distribution of T14 versus ISD patients in Southern Mexico, 2008-2010. Patients recruited to T14 (in dark blue) or ISD (in light blue). An arbitrary division (red line) separates areas according to the proportion of T14 vs ISD patients; a total of 59 ISD and 22 T14 patients came from the foothills (Z1; n = 81), and 8 ISD and 64 T14 patients came from Tapachula city and its surrounding areas (Z2; n = 72). Similar numbers of patients were recruited from both zones.
10.1186/s12936-015-0938-2 *Plasmodium vivax* genotypes from two groups of patients treated with combined chloroquine and primaquine by the intermittent single dose (ISD) and 14-day (T14) regimens.
10.1186/s12936-015-0938-2 Comparison of the *Plasmodium vivax* recurrent blood infection in T14 and ISD patients by geographic location.
10.1186/s12936-015-0938-2 Comparison of the cumulative incidence of *Plasmodium vivax* recurrences in patients receiving T14 (CQ-PQ); supervised (T14) and semi-supervised (T14 s), and followed up for ~12-months.
10.1186/s12936-015-0938-2 Parameters of primary and recurrent *Plasmodium vivax* blood infections for patients of the ISD group.

## Abbreviations

CQ: chloroquine
PQ: primaquine
ACT: artemisinin-based combination therapy
AHA: acute haemolytic anaemia
API: annual parasite index
T14: radical treatment of 14 days
ISD: intermittent single dose regimen
G6PDd: glucose 6-phosphate dehydrogenase deficiency
bw: body weight
MCP: malaria control programme
CRISP: Regional Centre for Public Health Research-INSP, Mexico
PCR: polymerase chain reaction
RDT: rapid diagnostic test
WBC: white blood cells
ELISA: enzyme linked immunosorbent assay
RFLP: restriction fragment length polymorphism
*cspr*: circumsporozoite gene central repeat
*msp3α*: merozoite surface protein 3α gene
*msp3β*: merozoite surface protein 3β gene
TF: therapeutic failure
ACPR: adequate clinical and parasitological response
IQR: interquartile range

## References

[1] Boletín Epidemiológico 1995–2005, Ministry of Health. Mexico. 2005. http://www.epidemiologia.salud.gob.mx/dgae/boletin/intd_boletin.html. Accessed 30 Sep 2015.

[2] Report on the situation of malaria in the Americas. Pan American Health Organization, Washington DC. 2011. http://www.paho.org/hq/index.php?option=com_content&view=article&id=5175%3A2010-report-on-situation-malaria-americas-2011&catid=1841%3Amalaria-publications&Itemid=40360&lang=en. Accessed 30 Sep 2015.

[3] Rodriguez MH, Betanzos-Reyes AF (2011). Grupo de Trabajo de Malaria del Sistema Mesoamericano de Salud Pública: plan de mejoramiento del control de la malaria hacia su eliminación en México. Salud Publica Mex.

[4] WHO. World malaria report 2012. World Health Organization, Geneva. 2012. http://www.who.int/malaria/publications/world_malaria_report_2012/eng/. Accessed 30 Sep 2015.

[5] Gilles HM, Warrell DA, Bruce-Chwatt LJ (1993). Bruce-Chwatt’s essential malariology.

[6] Coatney GR (1963). Pitfalls in a discovery: the chronicle of chloroquine. Am J Trop Med Hyg.

[7] White NJ (1998). Drug resistance in malaria. Br Med Bull.

[8] WHO. Guidelines for the treatment of malaria. World Health Organization, Geneva. 2015. http://www.who.int/malaria/publications/atoz/9789241549127/en/. Accessed 30 Sep 2015.

[9] Galappaththy GN, Omari AA, Tharyan P (2007). Primaquine for preventing relapses in people with *Plasmodium vivax* malaria. Cochrane Database Syst Rev..

[10] Norma Oficial Mexicana para la vigilancia epidemiológica, prevención y diagnóstico de enfermedades transmitidas por vector (NOM-032-SSA2-2002). Ministry of Health, Mexico. 2002. http://www.salud.gob.mx/unidades/cdi/nom/032ssa202.html. Accessed 30 Sep 2015.

[11] Arámbula E, Aguilar LJC, Vaca G (2000). Glucose-6-phosphate dehydrogenase mutations and haplotypes in Mexican mestizos. Blood Cells Mol Dis.

[12] Vaca G, Arámbula E, Esparza A (2002). Molecular heterogeneity of glucose-6-phosphate dehydrogenase deficiency in Mexico: overall results of a 7-year project. Blood Cells Mol Dis.

[13] Monteiro WM, Val FFA, Siqueira AM, Franca GP, Sampaio VS, Melo GC (2014). G6PD deficiency in Latin America: systematic review on prevalence and variants. Mem Inst Oswaldo Cruz.

[14] Bergonzoli G, Rivers Cuadra JC (2000). Therapeutic efficacy of different antimalarial regimens in the Costa Rica-Nicaragua border region. Rev Panam Salud Publica.

[15] Baird JK, Basry H, Subianto B, Fryaudff DJ, McElroy PD, Leksana B (1995). Treatment of chloroquine-resistant *Plasmodium vivax* with chloroquine and primaquine or halofantrine. J Infect Dis.

[16] Chanon KE, Mendez-Galván JF, Galindo-Jaramillo JM, Olguin-Bernal H, Borja-Aburto VH (2003). Cooperative actions to achieve malaria control without the use of DDT. Int J Hyg Environ Health.

[17] Mendez Galvan JF, Guerrero Alvarado J, González Mora M, PerezLanda M, Quintero-Cabanillas R (1984). Evaluation of an alternative scheme of treatment for the control of malaria. Salud Publica Mex..

[18] Bray PG, Deed S, Fox E, Kalkanidis M, Mungthin M, Deady LW (2005). Primaquine synergises the activity of chloroquine against chloroquine-resistant *P. falciparum*. Biochem Pharmacol.

[19] Murphy GS, Basri H, Purnomo Andersen EM, Bangs MJ, Mount DL (1991). Vivax malaria resistant to treatment and prophylaxis with chloroquine. Lancet.

[20] Gonzalez-Ceron L, Mu J, Santillan F, Joy D, Sandoval MA, Camas G (2013). Molecular and epidemiological characterization of *Plasmodium vivax* recurrent infections in southern Mexico. Parasit Vectors..

[21] The malERA consultative Group on Drugs (2011). A research agenda for malaria eradication: drugs. PLoS Med.

[22] Guía práctica revisada para estudios de eficacia de los medicamentos antimaláricos en las Américas. Pan American Health Organization. 2010. http://www.usaidami.org/extras/modificada-PAN-JUL-2010-integrada-2011.pdf. Accessed 30 Sep 2015.

[23] Ruebush TK, Marquino W, Zegarra J, Neyra D, Villaroel R, Avila JC (2003). Practical aspects of in vivo antimalarial drug efficacy testing in the Americas. Am J Trop Med Hyg.

[24] Gonzalez-Ceron L, Rodriguez MH, Betanzos AF, Abadia A (2005). Efficacy of a rapid test to diagnose *Plasmodium vivax* in symptomatic patients of Chiapas, Mexico. Salud Publica Mex..

[25] WHO. Methods for surveillance of antimalarial drug efficacy. World Health Organization, Geneva. 2009. http://www.who.int/malaria/publications/atoz/9789241597531/en/. Accessed 30 Sep 2015.

[26] Cucunubá ZM, Guerra AP, Rahirant SJ, Rivera JA, Cortés LJ, Nicholls RS (2008). Asymptomatic *Plasmodium spp*. infection in Tierralta, Colombia. Mem Inst Oswaldo Cruz.

[27] Fru-Cho J, Bumah VV, Safeukui I, Nkuo-Akenji T, Titanji VP, Haldar K (2014). Molecular typing reveals substantial *Plasmodium vivax* infection in asymptomatic adults in a rural area of Cameroon. Malar J..

[28] Rubio JM, Benito A, Roche J, Berzosa PJ, Garcia ML, Micó M (1999). Semi-nested, multiplex polymerase chain reaction for detection of human malaria parasites and evidence of *Plasmodium vivax* infection in Equatorial Guinea. Am J Trop Med Hyg.

[29] Hornstein JH, Miller MJ, Thiara S (1983). The indirect immunofluorescence (IIF) test in detection of imported *Plasmodium vivax* malaria in the Sutter-Yuba County area of California, U.S.A., 1975–1979. Am J Trop Med Hyg.

[30] Park JW, Moon SH, Yeom JS, Lim KJ, Sohn MJ, Jung WC (2001). Naturally acquired antibody responses to the C-terminal region of merozoite surface protein 1 of *Plasmodium vivax* in Korea. Clin Diagn Lab Immunol.

[31] Torres KJ, Clark EH, Hernandez JN, Soto-Cornejo KE, Gamboa D, Branch OH (2008). Antibody response dynamics to the *Plasmodium falciparum* conserved vaccine candidate antigen, merozoite surface protein-1 C-terminal 19kD (MSP1-19kD), in Peruvians exposed to hypoendemic malaria transmission. Malar J..

[32] Gonzalez-Ceron L, Rodriguez MH (1991). An enzyme-linked immunosorbent assay using detergent-soluble *Plasmodium vivax* antigen for seroepidemiological surveys. Trans R Soc Trop Med Hyg.

[33] Yang Z, Miao J, Huang Y, Li X, Putaporntip C, Jongwutiwes S (2006). Genetic structures of geographically distinct *Plasmodium vivax* populations assessed by PCR/RFLP analysis of the merozoite surface protein 3beta gene. Acta Trop.

[34] Cedillos RA, Warren M, Jeffery GM (1978). Field evaluation of primaquine in the control of *Plasmodium vivax*. Am J Trop Med Hyg.

[35] Lee KS, Kim TH, Kim ES, Lim HS, Yeom JS, Jun G (2009). Short report: chloroquine-resistant *Plasmodium vivax* in the Republic of Korea. Am J Trop Med Hyg.

[36] Castillo CM, Osorio LE, Palma GI (2002). Assessment of therapeutic response of *Plasmodium vivax* and *Plasmodium falciparum* to chloroquine in a malaria transmission free area in Colombia. Mem Inst Oswaldo Cruz.

[37] Graf PC, Durand S, Alvarez Antonio C, Montalvan C, Galves Montoya M, Green MD (2012). Failure of supervised chloroquine and primaquine regimen for the treatment of *Plasmodium vivax* in the Peruvian Amazon. Malar Res Treat..

[38] Mishra N, Singh JP, Srivastava B, Arora U, Shah NK, Ghosh SK (2012). Monitoring antimalarial drug resistance in India via sentinel sites: outcomes and risk factors for treatment failure, 2009–2010. Bull World Health Organ.

[39] Leslie T, Mayan I, Mohammed N, Erasmus P, Kolaczinski J, Whitty CJ (2008). A randomised trial of an eight-week, once weekly primaquine regimen to prevent relapse of *Plasmodium vivax* in Northwest Frontier Province, Pakistan. PLoS One..

[40] Liu H, Yang HL, Xu JW, Wang JZ, Nie RH, Li CF (2013). Artemisinin-naphthoquine combination versus chloroquine-primaquine to treat vivax malaria: an open-label randomized and non-inferiority trial in Yunnan Province, China. Malar J..

[41] Muhamad P, Chacharoenkul W, Rungsihirunrat K, Ruengweerayut R, Na-Bangchang K (2011). Assessment of in vitro sensitivity of *Plasmodium vivax* fresh isolates. Asian Pac J Trop Biomed..

[42] Awab GR, Pukrittayakamee S, Imwong M, Dondorp AM, Woodrow CJ, Lee SJ (2010). Dihydroartemisinin-piperaquine versus chloroquine to treat *vivax* malaria in Afghanistan: an open randomized, non-inferiority, trial. Malar J..

[43] Kinzer MH, Chand K, Basri H, Lederman ER, Susanti AI, Elyazar I (2010). Active case detection, treatment of *falciparum* malaria with combined chloroquine and sulphadoxine/pyrimethamine and *vivax* malaria with chloroquine and molecular markers of anti-malarial resistance in the Republic of Vanuatu. Malar J..

[44] Heidari A, Keshavarz H, Shojaee S, Raeisi A, Dittrich S (2012). In vivo susceptibility of *Plasmodium vivax* to chloroquine in Southeastern Iran. Iran J Parasitol..

[45] Teka H, Petros B, Yamuah L, Tesfaye G, Elhassan I, Muchohi S (2008). Chloroquine-resistant *Plasmodium vivax* malaria in Debre Zeit, Ethiopia. Malar J..

[46] Ould Ahmedou Salem MS, Mohamed Lemine YO, Deida JM, Lemrabott MA, Ouldabdallahi M, Ba MD (2015). Efficacy of chloroquine for the treatment of *Plasmodium vivax* in the Saharan zone in Mauritania. Malar J..

[47] Adelusi SA, Dawodu AH, Salako LA (1982). Kinetics of the uptake and elimination of chloroquine in children with malaria. Br J Clin Pharmacol.

[48] Adak T, Sharma VP, Orlov VS (1998). Studies on the *Plasmodium vivax* relapse pattern in Delhi, India. Am J Trop Med Hyg..

[49] Contacos PG, Collins WE, Jeffery GM, Krotoski WA, Howard WA (1972). Studies on the characterization of *Plasmodium vivax* strains from Central America. Am J Trop Med Hyg.

[50] Beutler E (2008). Glucose-6-phosphate dehydrogenase deficiency: a historical perspective. Blood.

[51] Cappellini MD, Fiorelli G (2008). Glucose-6-phosphate dehydrogenase deficiency. Lancet.

[52] Howes RE, Battle KE, Satyagraha AW, Baird JK, Hay SI (2013). G6PD deficiency: global distribution, genetic variants and primaquine therapy. Adv Parasitol.

[53] Domingo GJ, Satyagraha AW, Anvikar A, Baird K, Bancone G, Bansil P (2013). G6PD testing in support of treatment and elimination of malaria: recommendations for evaluation of G6PD tests. Malar J..

[54] Bussaratid V, Walsh DS, Wilairatana P, Krudsood S, Silachamroon U, Looareesuwan S (2000). Frequency of pruritus in *Plasmodium vivax* malaria patients treated with chloroquine in Thailand. Trop Doct.

[55] Ballut PC, Siqueira AM, Orlando AC, Alexandre MA, Lacerda MV (2013). Prevalence and risk factors associated to pruritus in *Plasmodium vivax* patientes using chloroquine in the Brazilian Amazon. Acta Trop.

[56] Pinto AY, Azevedo CH, da Silva JB, de Souza JM (2003). Assessment of chloroquine single dose treatment of malaria due to *Plasmodium vivax* in Brazilian Amazon. Rev Inst Med Trop Sao Paulo.

[57] Delgado-Ratto C, Soto-Calle VE, Van den Eede P, Gamboa D, Rosas A, Abatih EN (2014). Population structure and spatio-temporal transmission dynamics of *Plasmodium vivax* after radical cure treatment in a rural village of the Peruvian Amazon. Malar J..

[58] Baird JK (2004). Chloroquine resistance in *Plasmodium vivax*. Antimicrob Agents Chemother.

[59] Baird JK, Leksana B, Masbar S, Fryauff DJ, Sutanihardja MA, Suradi (1997). Diagnosis of resistance to chloroquine by *Plasmodium vivax*: timing of recurrence and whole blood chloroquine levels. Am J Trop Med Hyg.

[60] Hanf M, Stephani A, Basurko C, Nacher M, Carme B (2009). Determination of the Plasmodium vivax relapse pattern in Camopi, French Guiana. Malar J..

[61] White NJ (2011). Determinants of relapse periodicity in *Plasmodium vivax* malaria. Malar J..

[62] Congpuon K, Satimai W, Sujariyakul A, Intanakom S, Harnpitakpong W, Pranuth Y (2011). In vivo sensitivity monitoring of chloroquine for the treatment of uncomplicated *vivax* malaria in four bordered provinces of Thailand during 2009–2010. J Vector Borne Dis..

[63] Ducharme J, Farinotti R (1996). Clinical pharmacokinetics and metabolism of chloroquine. Focus on recent advancements. Clin Pharmacokinet..

[64] Pussard E, Verdier F (1994). Antimalarial 4-aminoquinolines: mode of action and pharmacokinetics. Fundam Clin Pharmacol.

[65] Rodriguez MH, Knols BGJ, Louis C (2006). Malaria and dengue vector biology and control in Latin America. Bridging laboratory and field research for genetic control of disease vectors.

[66] Gomez MI. Comparative study of two regimens of radical treatment of vivax malaria in Mexico. World Health Organization, Geneva. 1965. http://www.who.int/iris/handle/10665/65295. Accessed 30 Sep 2015.

[67] Blair S, Giraldo C (1991). Reinfección endógena por Plasmodium vivax. IATREIA..

[68] Carmona-Fonseca J, Maestre A (2009). Prevention of *Plasmodium vivax* malaria recurrence: efficacy of the standard total dose of primaquine administered over 3 days. Acta Trop.

[69] Alvarez G, Pineros JG, Tobon A, Rios A, Maestre A, Blair S (2006). Efficacy of three chloroquine-primaquine regimens for treatment of *Plasmodium vivax* malaria in Colombia. Am J Trop Med Hyg.

[70] Schwartz E, Regev-Yochay G, Kurnik D (2000). Short report: a consideration of primaquine dose adjustment for radical cure of *Plasmodium vivax* malaria. Am J Trop Med Hyg.

[71] Ingram RJ, Crenna-Darusallam C, Soebianto S, Noviyanti R, Baird JK (2014). The clinical and public health problem of relapse despite primaquine therapy:case review of repeated relapses of *Plasmodium vivax* acquired in Papua New Guinea. Malar J..

[72] Alonso PL, Brown G, Arevalo-Herrera M, Binka F, Chitnis C, Collins F (2011). A research agenda to underpin malaria eradication. PLoS Med..

