# Supplementary material for: Effectiveness of combined chloroquine and primaquine treatment in 14 days versus intermittent single dose regimen, in an open, non-randomized, clinical trial, to eliminate Plasmodium vivax in southern Mexico
Source: Malar J. 2015 Oct 30;14:426. doi: 10.1186/s12936-015-0938-2 (PMC4628368; doi:10.1186/s12936-015-0938-2)
Supplement: Supplementary file 1 — 10.1186/s12936-015-0938-2 Plasmodium vivax treatment regimens based on chloroquine and primaquine, by age groups as indicated in the Mexican guidelines for malaria treatment. [file 12936_2015_938_MOESM1_ESM.pdf]

Additional file 1 ***Plasmodium vivax*** **treatment regimens based on chloroquine and primaquine, by age groups as indicated in the Mexican guidelines for malaria treatment**

Table 8. Treatment with intermittent single combined doses; ISD

Age group:	No. of tablets of chloroquine, 150 mg	No. of tablets of primaquine, 5mg	No. of tablets of primaquine, 15mg
1 year old	1/2	1	0
2 a 5 years old	1	2	0
6 a 12 years old	2	4	0
13 years old and over:<br>about 60 kg of body weight	3	0	2
above 60 kg of body weight	4	0	3

Table 10. Treatment for radical cure of 14 days; T14

Age group:	*No. of tablets of chloroquine, 150 mg		No. of tablets of primaquine, daily during 14 days	
	First day	2nd and 3th Days	5 mg	15 mg
1 year old	1	1/2	1/2	0
2 a 5 years old	1,1/4	1	1	0
6 a 12 years old	2	1,1/2	2	0
13 years old and over:<br>about 60 kg of body weight	3	2, 1/4	0	1
above 60 kg of body weight	4	3	0	1

*adjusted to administer for three days

NOM-032-SSA2-2002, Available at:
http://www.salud.gob.mx/unidades/cdi/nom/032ssa202.html.
