# Supplementary material for: Effectiveness of combined chloroquine and primaquine treatment in 14 days versus intermittent single dose regimen, in an open, non-randomized, clinical trial, to eliminate Plasmodium vivax in southern Mexico
Source: Malar J. 2015 Oct 30;14:426. doi: 10.1186/s12936-015-0938-2 (PMC4628368; doi:10.1186/s12936-015-0938-2)
Supplement: Supplementary file 2 — 10.1186/s12936-015-0938-2 Chloroquine and primaquine drug batches used during the study. [file 12936_2015_938_MOESM2_ESM.pdf]

Additional file 2 Chloroquine and primaquine drug batches used during the study

Laboratory	Code	Drugs	Batch	Expiration date	mg per tablet	Period of distribution
Proteín S.A C.V	2030	Chloroquine phosphate	7112877	November 2009	150	2008-2009
Probiomed S.A de C.V	2030	Chloroquine phosphate	2030060709	July 2009	150	2008-2009
Proteín S.A de C.V	2030	Chloroquine phosphate	7K2265	November 2012	150	2009-2010
WEIFA AS, OSLO NORUEGA		Primaquine phosphate	0731629	September 2010	15	2009
Proteín S.A de C.V	2032	Primaquine phosphate	6A0239	September 2009	15	2008
Proteín S.A de C.V	2032	Primaquine phosphate	8B0555	May 2011	15	2010
Proteín S.A de C.V	2031	Primaquine phosphate	7H1633	August 2010	5	2009-2010
Proteín S.A de C.V	2031	Fosfato de primaquina	6C0547	March 2009	5	2008

The batches used were provided by the local malaria control program during the study.
The use of each lot was discontinued at the expiration date
