# Supplementary material for: Effectiveness of combined chloroquine and primaquine treatment in 14 days versus intermittent single dose regimen, in an open, non-randomized, clinical trial, to eliminate Plasmodium vivax in southern Mexico
Source: Malar J. 2015 Oct 30;14:426. doi: 10.1186/s12936-015-0938-2 (PMC4628368; doi:10.1186/s12936-015-0938-2)
Supplement: Supplementary file 3 — 10.1186/s12936-015-0938-2 Study site and geographic distribution of T14 versus ISD patients in Southern Mexico, 2008-2010. Patients recruited to T14 (in dark blue) or ISD (in light blue). An arbitrary division (red line) separates areas according to the proportion of T14 vs ISD patients; a total of 59 ISD and 22 T14 patients came from the foothills (Z1; n = 81), and 8 ISD and 64 T14 patients came from Tapachula city and its surrounding areas (Z2; n = 72). Similar numbers of patients were recruited from both zones. [file 12936_2015_938_MOESM3_ESM.pdf]

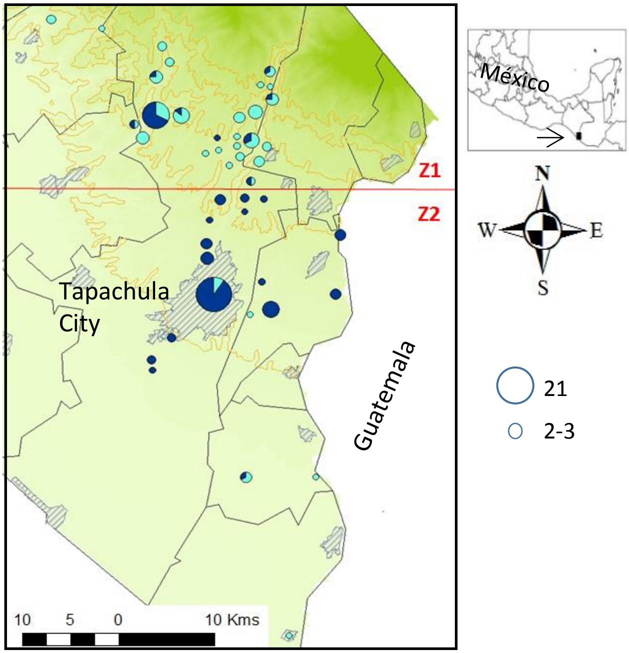

Additional file 3 Study site and geographic distribution of T14 versus ISD patients in Southern Mexico, 2008-2010

Patients recruited to T14 (in dark blue) or ISD (in light blue). An arbitrary division (red line) separates areas according to the proportion of T14 vs ISD patients; a total of 59 ISD and 22 T14 patients came from the foothills (Z1; n=81), and 8 ISD and 64 T14 patients came from Tapachula city and its surrounding areas (Z2; n=72). Similar numbers of patients were recruited from both zones.
