# Supplementary material for: Effectiveness of combined chloroquine and primaquine treatment in 14 days versus intermittent single dose regimen, in an open, non-randomized, clinical trial, to eliminate Plasmodium vivax in southern Mexico
Source: Malar J. 2015 Oct 30;14:426. doi: 10.1186/s12936-015-0938-2 (PMC4628368; doi:10.1186/s12936-015-0938-2)
Supplement: Supplementary file 4 — 10.1186/s12936-015-0938-2 Plasmodium vivax genotypes from two groups of patients treated with combined chloroquine and primaquine by the intermittent single dose (ISD) and 14-day (T14) regimens. [file 12936_2015_938_MOESM4_ESM.pdf]

Additional file 4 *Plasmodium vivax* genotypes from two groups of patients treated with combined chloroquine and primaquine by the intermittent single dose (ISD) and 14-day (T14) regimens

**RFLP genotype:**	**Treatment regimen (CQ-PQ):**	
cspr-msp3α	ISD	14-day
vk247-A	15	18
vk247-B	4	4
vk247-C	0	3
Vk210-A	0	1
vk210-B	12	23
vk210-C	21	23
vk210-g	1	2
vk210-K	1	6
vk210-F	1	0
vk210-E	2	1
vk210-D	1	2
Mixed genotypes:	7*	3**
ND	2	0
Total	67	86

RFPL, restriction fragment length polymorphism; CQ, chloroquine; PQ, primaquine; * mixed genotype infections: five with *cspr* (vk210-247) and *msp3α* (A, n=3; B, n=1; C, n=1); two with vk247 and *msp3α* (AB and AC); ND, not determined (one sample did not amplify msp3α /and the other sample was not available for PCR analysis); ** All were mixed genotype infections, with *cspr* and *msp3α* –A; no differences in the proportions of genotypes between treatment groups were detected, with Fisher's exact test (α=0.05), p=0.248
