# Supplementary material for: Effectiveness of combined chloroquine and primaquine treatment in 14 days versus intermittent single dose regimen, in an open, non-randomized, clinical trial, to eliminate Plasmodium vivax in southern Mexico
Source: Malar J. 2015 Oct 30;14:426. doi: 10.1186/s12936-015-0938-2 (PMC4628368; doi:10.1186/s12936-015-0938-2)
Supplement: Supplementary file 5 — 10.1186/s12936-015-0938-2 Comparison of the Plasmodium vivax recurrent blood infection in T14 and ISD patients by geographic location. [file 12936_2015_938_MOESM5_ESM.pdf]

Additional file 5 Comparison of the *Plasmodium vivax* recurrent blood infection in T14 and ISD patients by geographic location

Zones:	Treatment: T14		ISD	
	Patients, n	W/recurrent infection (%)	Patients, n	W/recurrent infection (%)
Z1, foothills	9	0 (0)	43	16 (37.2)
Z2, Tapachula city and its surroundings	28	4 (14.2)	6	2 (33.3)
	37	*p=0.310*	49	*p=0.616*

n, number of patients under follow up; Fisher exact test at 95% CI (see also additional file 3).
