# Supplementary material for: Effectiveness of combined chloroquine and primaquine treatment in 14 days versus intermittent single dose regimen, in an open, non-randomized, clinical trial, to eliminate Plasmodium vivax in southern Mexico
Source: Malar J. 2015 Oct 30;14:426. doi: 10.1186/s12936-015-0938-2 (PMC4628368; doi:10.1186/s12936-015-0938-2)
Supplement: Supplementary file 6 — 10.1186/s12936-015-0938-2 Comparison of the cumulative incidence of Plasmodium vivax recurrences in patients receiving T14 (CQ-PQ); supervised (T14) and semi-supervised (T14 s), and followed up for ~12-months. [file 12936_2015_938_MOESM7_ESM.pdf]

Additional file 7 Parameters of primary and recurrent *Plasmodium vivax* blood infections for patients of the ISD group

Patient	Age/ sex	Primary blood infection: (p/µl) asexual parasitaemia	Primary blood infection: RFLP-genotype: *cspr-msp3α/β*	Recurrent infection by microscopy: [a]Day	clinical symptoms:	(p/µl) asexual parasitaemia	RFLP-genotype: *cspr-msp3α/β*	Serology (ELISA OD value/ 405NM; cut off value 0.25)
P6	3/F	2,364	vk247-A	[b]**D241**	Yes	875	vk247-A	0.16_{D211}→2.57_{D241}
P7	17/F	10,766	vk210-B/III	[b]**D236**	Yes	14,218	vk210-B/III	0.04_{D218}→1.49_{D236}
P15	62/F	675	vk210-B/IV	[b]D312	Yes	246	vk210-B/IV	0.29_{D301}→ $>3.0_{D312}$
P17	36/F	480	vk210/247-B/III	[b]D108	Yes	8,485	vk247-A/I	0.18_{D89}→ 2.1_{D209}
P35	20/F	2,241	vK210-C/V	[b]D336	Yes	846	vK210-C/V	0.18_{D308}→ 1.88_{D336}
P37	9/M	17,598	vK210-C/V	[b]D287	Yes	866	vK210-C/V	0.16_{D287} → 1.67_{D308}
P39*	2/M	4,628	vk210/247-B/IV	[c]**D209**	Yes	Mc-PCR-		0.17_{D134} → 1.75_{D209}
				[c]**D229**	No	Mc-PCR-		→ 1.07_{D229}
				D259	No	Mc-PCR-		→ 0.93_{D259}
				[b]D287	Yes	5,068	vk247-B/IV	→ 1.82_{D287}
P44	60/M	2,951	vk210-C/VI	[b]D282	Yes	844	vk210-C/VI	0.18_{D244} → 0.53_{D282}
P45	63/M	496	vk247-A	[c]**D248**	No	Mc-PCR+	-	0.3_{D213}→$.547_{D248}$
				D271	No	Mc-PCR-		→1.34_{D271}
				[b]D297[1]	Yes	205	vk247-A	→2.29_{D297}

P55	32/F	**3,171**	vk210-g	[c]**D31**	No	56	vK210-g	-
				[b]D152	Yes	182	vk210/247-g/A	$0.36_{D122} \rightarrow 1.32_{D152}$
P56	42/F	**7,960**	vk210-C/V	[b]**D178**	Yes	1,570	vk210/247-A/I	$0.41_{D147} \rightarrow 1.12_{D178}$
P62	26/M	4,319	vk210/247-C/II	[b]**D216**	Yes	2,594	vk210-C/II	$0.15_{D183} \rightarrow 2.48_{D216}$
P63	49/M	640	ND	[c]**D74**	Yes	Mc-PCR+	vk210-B/III	$0.74_{D57} \rightarrow 1.83_{D74}$
				[b]D183	Yes	6,238	vk210-B/III	$0.554_{D161} \rightarrow 2.09_{D183}$
P71*	6/F	9,294	vk210-C/V	[c]**D32**	No	343	vk210-C/V	increased at D90 and after
				[c]**D66**	No	347	vk210-C/V	
P72	12/M	8,059	vk210-B/III	D98[2]	Yes	3,505	vk210-B/III	$0.963_{D92} \rightarrow 2.03_{D98}$
P80*	1/M	8,413	vk247-A	[c]**D210**	No	Mc-PCR-	-	$0.25_{D189} \rightarrow 1.71_{D210}$
				[c]**D252**	No	4,603	vk247-A	$\rightarrow 0.84_{D252}$
				[b]D292	No	529	ND	$\rightarrow 1.19_{D292}$
P81*	6/F	5,106	vk210-C/V	[b]D95	Yes	1,378	vk210-C/V	$0.18_{D91} \rightarrow 0.59_{D95}$
P136	9/M	5,744	vk210-C/V	[b]D352*	Yes	10,029	vk210-C/V	$0.12_{D338} \rightarrow 1.31_{D352}$

M, male; F, female; RFLP, restriction fragment length polymorphism; *cspr*, circumsporozoite repeat; *msp3α*, merozoíte surface protein 3α and 3β; [a] day of visit, and in bold, the day that ISD was scheduled (b, after diagnosed with *P. vivax*, T14 was administered; c, continued the ISD treatment); *difficult to register other symptoms; ND, not determined; Mc, microscopy; three patients were detected with an antibody increase only (P22, $0.186_{D315} \rightarrow 0.57_{D341}$, P87, $0.55_{D121} \rightarrow 1.73_{D149}$, P115, $0.12_{D115} \rightarrow 0.90_{D158}$), in whom a parasite was not demonstrated by microscopy or PCR. P44, after T14 treatment had a second recurrent episode on D348 that was positive by microscopy; P59, a recurrent episode by microscopy was detected on D419, recorded by the malaria control programme
