# Supplementary material for: Effectiveness of combined chloroquine and primaquine treatment in 14 days versus intermittent single dose regimen, in an open, non-randomized, clinical trial, to eliminate Plasmodium vivax in southern Mexico
Source: Malar J. 2015 Oct 30;14:426. doi: 10.1186/s12936-015-0938-2 (PMC4628368; doi:10.1186/s12936-015-0938-2)
Supplement: Supplementary file 7 — 10.1186/s12936-015-0938-2 Parameters of primary and recurrent Plasmodium vivax blood infections for patients of the ISD group. [file 12936_2015_938_MOESM6_ESM.pdf]

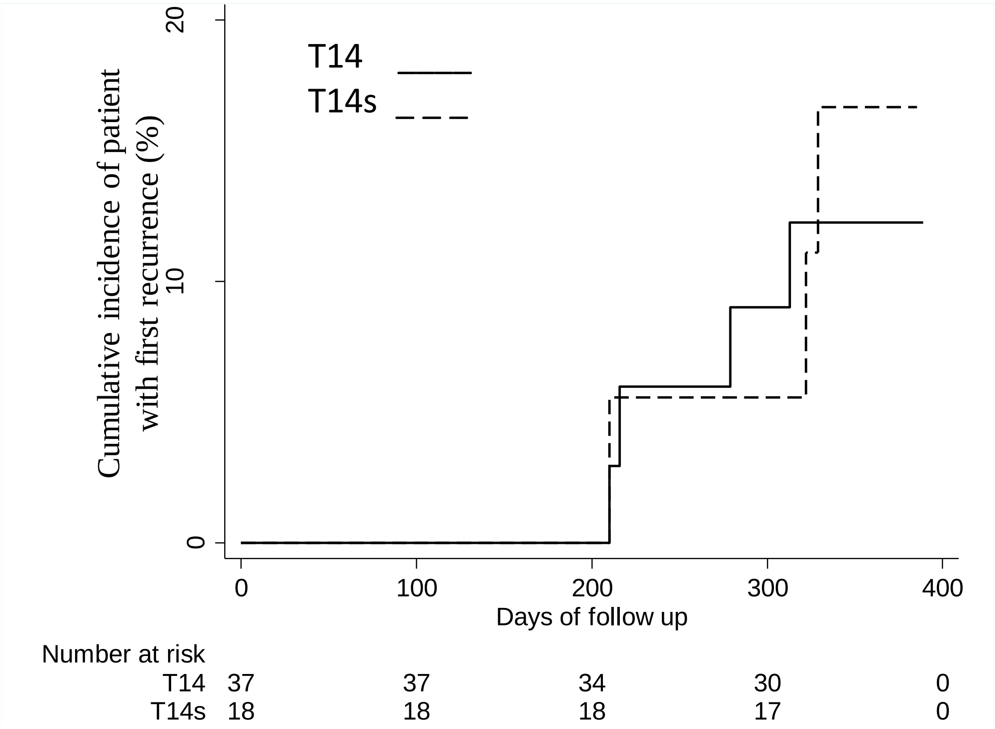

Additional file 6 Comparison of the cumulative incidence of *Plasmodium vivax* recurrences in patients receiving T14 (CQ-PQ); supervised (T14) and semi-supervised (T14s), and followed up for ~12 - months
